# AF10 Plays a Key Role in the Survival of Uncommitted Hematopoietic Cells

**DOI:** 10.1371/journal.pone.0051626

**Published:** 2012-12-19

**Authors:** Raquel Chamorro-Garcia, Margarita Cervera, Juan J. Arredondo

**Affiliations:** Departamento de Bioquímica & Instituto Investigaciones Biomédicas, The Autonomous University of Madrid-Consejo Superior de Investigaciones Científicas, Madrid, Spain; Emory University, United States of America

## Abstract

Hematopoiesis is a complex process regulated by both cell intrinsic and cell extrinsic factors. Alterations in the expression of critical genes during hematopoiesis can modify the balance between stem cell differentiation and proliferation, and may ultimately give rise to leukemia and other diseases. AF10 is a transcription factor that has been implicated in the development of leukemia following chromosomal rearrangements between the AF10 gene and one of at least two other genes, *MLL* and *CALM*. The link between AF10 and leukemia, together with the known interactions between AF10 and hematopoietic regulators, suggests that AF10 may be important in hematopoiesis and in leukemic transformation. Here we show that AF10 is important for proper hematopoietic differentiation. The induction of hematopoietic differentiation in both human hematopoietic cell lines and murine total bone marrow cells triggers a decrease of AF10 mRNA and protein levels, particularly in stem cells and multipotent progenitors. Gain- and loss-of-function studies demonstrate that over- or under-expression of AF10 leads to apoptotic cell death in stem cells and multipotent progenitors. We conclude that AF10 plays a key role in the maintenance of multipotent hematopoietic cells.

## Introduction

In adult vertebrates, hematopoiesis takes place in the bone marrow starting with the integration of signals originated by cells located in the hematopoietic stem cell (HSC) environment, known as the HSC niche. These surrounding cells secrete specific molecules, such as cytokines and chemokines, that initiate and promote stem cell proliferation and differentiation and provide an extracellular matrix that serves as an anchoring point for adhesion of HSCs [1]. Daughter cells generated from HSCs can either retain their self-renewal capability or may become committed to a cell lineage and subsequently undergo terminal differentiation [1], [2]. Upon the integration of extracellular signals, the expression of key genes in immature hematopoietic cells is modified, switching on specific pathways that result in the change of the characteristics of the cells [3], [4]. Aberrant expression of these key genes during hematopoiesis may lead to the development of different hematological diseases, such as leukemia [5].

Chromosomal rearrangement of genes encoding transcription factors with important roles during hematopoiesis are frequently associated with leukemic processes [6]. One such frequently-rearranged genes is *MLL* (Mixed Lineage Leukemia), located at 11q23, which encodes a highly conserved transcription factor belonging to the Trithorax family of transcriptional activators [7]. MLL has been characterized as a major regulator of the homeotic group of genes (*Hox* genes) [7] as well as a key controller of hematopoiesis [8], [9], [10]. *MLL* is implicated in at least 70 different chromosomal translocations associated with the development of both infant and adult leukemias [11], [12], [13], [14], [15]. However, despite the broad range of partners, 85% of the described translocations involve rearrangements that affect just six genes: *AF4, AF9, ENL, AF6, AF10* and *ELL*, which have been suggested to participate, together with MLL, in the same transcriptional regulator complex [15]. The *AF10* gene, located at 10p12, is the only one of these six *MLL* recurrent partners that has been found to participate in another leukemic translocation with the *CALM* gene (Clatrhin-Assembly Lymphoid-Myeloid) [16], [17], located at 11q14. CALM (also known as PICALM) is a cytoplasmic protein implicated in endocytosis [18]. The translocation t(10;11)(p12;q23) produces the MLL-AF10 fusion protein which is almost exclusively found in patients with acute myeloid leukemia [19]. Translocation t(10;11)(p12;q14) leads to the CALM-AF10 fusion protein, which is present in patients with acute myeloid or acute lymphoid leukemia [16]. Although AF10's target genes remain unknown, its implication in the development of myeloid, lymphoid and megakaryoblastic leukemia upon translocation with either *MLL* or *CALM* [16], [17] suggests that AF10 may be an important factor in the development of leukemia.

Although the molecular mechanisms through which AF10 influence the development of leukemia remain poorly understood, some regions of AF10, important for leukemic transformation, have been identified. In its C-terminal region, AF10 possesses an octapeptide motif followed by a leucine zipper domain (OM-LZ, Figure S1) implicated in its interaction with several proteins, including DOT1L [20], [21]. Molecular analyses of MLL- and CALM-AF10 rearrangements show differences in the location of AF10 breakpoints, but the OM-LZ domain is present in both fusion proteins (Figure S1) [16], [17], [20], [21]. Furthermore, Deshpande et al showed recently that the fusion between the clathrin binding domain from CALM and the OM-LZ from AF10 is sufficient to induce acute myeloid leukemia in mice [22]. DOT1L is a H3K79 histone-methyltransferase whose binding with AF10 modifies and activates the methylation pattern of the lysine 79 at histone H3 tails on target gene promoters [20], [21]. In the presence of MLL- or CALM-AF10 fusion proteins, aberrant increases in the expression level of genes such as *HoxA5*, *HoxA7*, *HoxA9*, *HoxA10* and *Meis1* have been described; all of these genes are involved in hematopoiesis [23]. AF10 has also been shown to interact with FLRG and Ikaros, both of which have been implicated in hematopoiesis [24], [25], [26]. FLRG participates in erythrocytic commitment, whereas Ikaros interacts with chromatin remodeling factors and plays a role in transcriptional regulation and cell cycle control during lymphocyte differentiation [27], [28], [29], [30]. AF10 was recently shown to be present in a complex also containing Tcf4/β-catenin and Dot1L in mouse small intestinal crypts, zebrafish, and Drosophila, where it participates in the maintenance of intestinal cell homeostasis [31], [32]. These data, together with the fact that AF10 interacts with other proteins implicated in the hematopoietic differentiation and its involvement in leukemic transformation [16], [17], suggest that AF10 has an important role in the regulation of cell proliferation and differentiation in several tissues.

We sought to further investigate the role of AF10 in hematopoiesis and leukemic transformation by testing the consequences of AF10 gain- or loss-of-function during hematopoietic differentiation. We utilized four different human cell lines (HEL, K562, CMK and HL-60) with the ability to differentiate through diverse hematopoietic pathways as well as immature primary hematopoietic stem cells obtained from mouse bone marrow to test the effects of modulating AF10 function. HEL cells follow either the megakaryocytic or the monocytic pathway, depending on the concentration of 12-O-tetradecanoylphorbol 13-acetate (TPA) used [33]. K562 and CMK cells are committed to the megakaryocytic lineage [34], [35], while HL60 cells follow the monocytic lineage [36]. The induction of differentiation in all four cells lines led to a decrease in both *AF10* mRNA and protein levels. Small variations in *AF10* gene expression induced apoptosis in HEL cells, the only one of the cell lines used capable to differentiate into diverse hematopoietic cell fates, but this effect was not observed in the cell lines with committed fate. Mouse bone marrow-derived immature hematopoietic cells show high AF10 expression levels, whereas these levels are very low in differentiated macrophages, recapitulating our observations in immortalized cell lines. Taken together, these data support the conclusion that AF10 plays an important role in the early stages of hematopoiesis but not in differentiated stages. These results provide insight into AF10 function during hematopoiesis and contribute to a better understanding of its role in leukemic transformation.

## Methods

All experiments were assayed in triplicates in at least three independent experiments. Figures show one representative independent experiment.

### Ethics statement

Mice used in the project were maintained according to the law (R.D. 1201/2005, October 10th, BOE, October 21st, 2005) and housed in the animal facility of UAM, Register number ES-28079-0000097, and approved by the Ethical committee for Research from the Universidad Autonoma de Madrid (Certificate Code C13–201). Mice were sacrificed with CO2 followed by cervical dislocation, a method that has been authorized by DG XI European Commission 2010/63/UE, published 22 September 2010, annex IV.

### Isolation of mouse primary cells from bone marrow

Bone marrow cells from 2–3 month-old CD1 mice were isolated by flushing the femurs and tibias with complete RPMI 1640 medium. Cells were filtered to obtain a single cell suspension, centrifuged at 1,000 RPM and cultured with complete RPMI 1640 medium at 37°C and 5% CO_2_. After 24 hours, cells were incubated in complete RPMI 1640 medium supplemented with 30% monocyte colony stimulation factor (M-CSF) for 7 days at 37°C and 5% CO_2_ to induce monocytic differentiation. M-CSF was obtained from the culture of L929 as described by Boltz-Nitulescu and collaborators [37]. Briefly, L929 cells were incubated with DMEM medium (Gibco) supplemented with 10% FBS (Gibco), 1% L-glutamine (Gibco) and 50 µg/ml gentamicine (Normon Laboratories S.A). Medium was changed when L929 cells were 90% confluent. After 72 hours with fresh media, medium was collected, filtered and kept frozen until use. The cell line L929 was a gift from Dr. Susana Alemany and its original source was ATCC.

### Cell culture and cell transfection

The cell lines HEL, K562, CMK and HL60 were grown in complete RPMI 1640 medium (RPMI 1640 medium (Invitrogen) supplemented with 10% FBS (Invitrogen), 1% L-glutamine (Invitrogen) and 50 µg/ml gentamicin (Normon Laboratories S.A)) at 37°C and 5% CO_2_. To induce megakaryocytic differentiation in HEL, K562 and CMK cells and monocytic differentiation in HL60 cells, cells were treated with 10 nM TPA (Sigma Aldrich) for 72 hours [33], [34], [35], [36]. HEL cells were differentiated through the monocytic pathway with 1 µM TPA for 72 hours [33]. For the inhibition of the proteosome, the HEL cell line was treated with 10 nM TPA during 24 h. After that period, the cells were exposed to 10 nM MG132 or DMSO (vehicle) together with the initial TPA treatment for 24 h more. For transient transfections, 10^6^ cells were transfected by nucleofection with the Amaxa® Cell Line Nucleofector® Kit V (Lonza) according to manufacturer's protocol in the Nucleofector® machine. Cells were cultured in complete RPMI 1640 medium with or without TPA (depending on the assay) at 37°C and 5% CO_2_ for 18 hours prior to their analysis. Cell lines HEL and K562 were kindly provided by Dr. Carmela Calés while CMK and HL60 were gifts from Dr. Miguel Angel Piris. The original source of these cell lines was ATCC.

### Quantitative RT-PCR analysis

Total RNA from cells was isolated with Trizol® Reagent (Invitrogen). The Quantitech Reverse Transcription Kit (Qiagen) was used for cDNA synthesis, according to the manufacturer protocol. Taqman Universal PCR Master Mix (Applied Biosystems) was used for q-RT-PCR to analyze *AF10* cDNA. The specific probes (Applied Biosystems) were Hs00946024_m1 for human *AF10* cDNA and Mm00487708_m1 for mouse *AF10* cDNA, and were used according to the manufacturer's recommendations. *AF10* expression was calculated for each cell type relative to 18S rRNA (Euk 18S rRNA) levels. The same 18S rRNA probe was used for human and mouse analyses.

### Western blot

5×10^5^ cells were lysed with 50 µl of lysis buffer (25 mM Tris-HCl pH 7.6, 150 mM NaCl, 1% NP-40, 1% sodium deoxycholate, 0.1% SDS and protease inhibitors) for 10 minutes on ice and centrifuged at 10,000 rpm for 10 min at 4°C. Protein concentration was measured with D_c_ Protein assay kit (Bio-Rad). 20 µg of protein were separated by SDS-PAGE electrophoresis and transferred to an Immobilon-PVDF membrane (Millipore). AF10 protein was detected with the monoclonal anti-AF10m (1∶1000; Abcam) or the polyclonal antibody anti-AF10p generated in the laboratory (1∶5000). Anti-TBP (1∶2000; Santa Cruz Biotechnology) was used as a control.

### Immunofluorescence

Cells adhered to coverslips coated with 20 µg/ml fibronectin (Sigma Aldrich) were fixed with 3.7% paraformaldehyde in PBS for 15 minutes. Cells were washed twice with PBS for 5 minutes and permeabilized and blocked in 5% Triton-X100 and 0.1 M glycine in PBS. Fixed cells were incubated overnight at 4°C with anti-AF10m antibody (1∶1000). After two 5 minute washes in PBS, cells were incubated with the appropriate secondary antibody for 1 hour in the dark and washed twice with PBS for 5 minutes. Nuclei were stained with 1 µg/ml DAPI (Invitrogen). Images were obtained using a Nikon Eclipse E400 microscope.

### Flow cytometry

For all the protocols described in this section, 5×10^5^ cells were washed with PBS and fixed with 10% formalin for 15 minutes in ice unless otherwise indicated. For detection of phosphatidylserine, cells were incubated with 3 µl of annexin V-DYE647 antibody (Immunostep) in 100 µl annexin-V buffer (10 mM Hepes pH 7.4, 140 mMNaCl, 2.5 mM CaCl_2_) for 15 minutes at room temperature in the dark. Annexin-V levels were determined as the proportion of Annexin-V positive cells present in the total GFP positive population. To detect the F4/80 or CD41 surface markers, cells were incubated with 3 µl of F4/80 or CD41 antibody (Serotec) in 100 µL PBS for 15 minutes at room temperature in the dark. For DNA content analyses, cells were fixed with cold 70% ethanol and kept at −20°C during 30 minutes. Cells were incubated with 50 µg/ml propidium iodide and 5 µg/ml RNAse in PBS. Cells were analyzed with a Beckman Coulter-FC 500 MLP cytometer and the CXP software.

### Constructs

For AF10 overexpression, KpnI and XbaI sites were terminally added to AF10 coding sequence by PCR using appropriate oligonucleotides. PCR products were then digested with both enzymes and cloned in the pcDNA4/TO plasmid (Invitrogen). GFP coding sequence was amplified from pStinger [38] and cloned in to pcDNA4/TO plasmid following the same strategy. This construct is referred to as GFP. To obtain the GFP-AF10 fusion protein-expressing construct, both cDNAs were amplified by PCR so that the GFP 3′ end overlaps with AF10 5′ end. Then, both fragments were used as bait in a second PCR round to obtain the GFP-AF10 cDNA. This construct is referred to as GFP-AF10. Sequences from these primers are available upon request. For iRNA experiments, BLOCK-iT™POL II miRRNAi Expression Vector Kit (Invitrogen) was used according to manufacturer's instructions. The scrambled control siRNA is referred to as siControl. AF10 mRNA interfering constructs are referred to as siAF10. Sequences from interfering harping are shown in Table S1.

### Statistical analyses

Data is presented as mean ± SEM. A paired t-test was used to determine the significance of the differences shown among treatments. *p*<0.05 was considered statistically significant. GraphPad Prism version 5.0 was used for statistical analyses.

## Results

### Expression pattern of AF10 during hematopoiesis

AF10 expression was assayed *in vitro* in a panel of four human cell lines (HEL, K562, CMK and HL60) representing different stages of hematopoietic differentiation. The HEL cell line, the most uncommitted cell line in the panel, can differentiate into megakaryocytes (when treated with 10 nM TPA) or into monocytes (1 µM TPA) [33]. K562 and CMK cells can differentiate into megakaryocytes and HL60 cells into monocytes when they are treated with 10 nM TPA [34], [35], [36].

**Figure 1:**
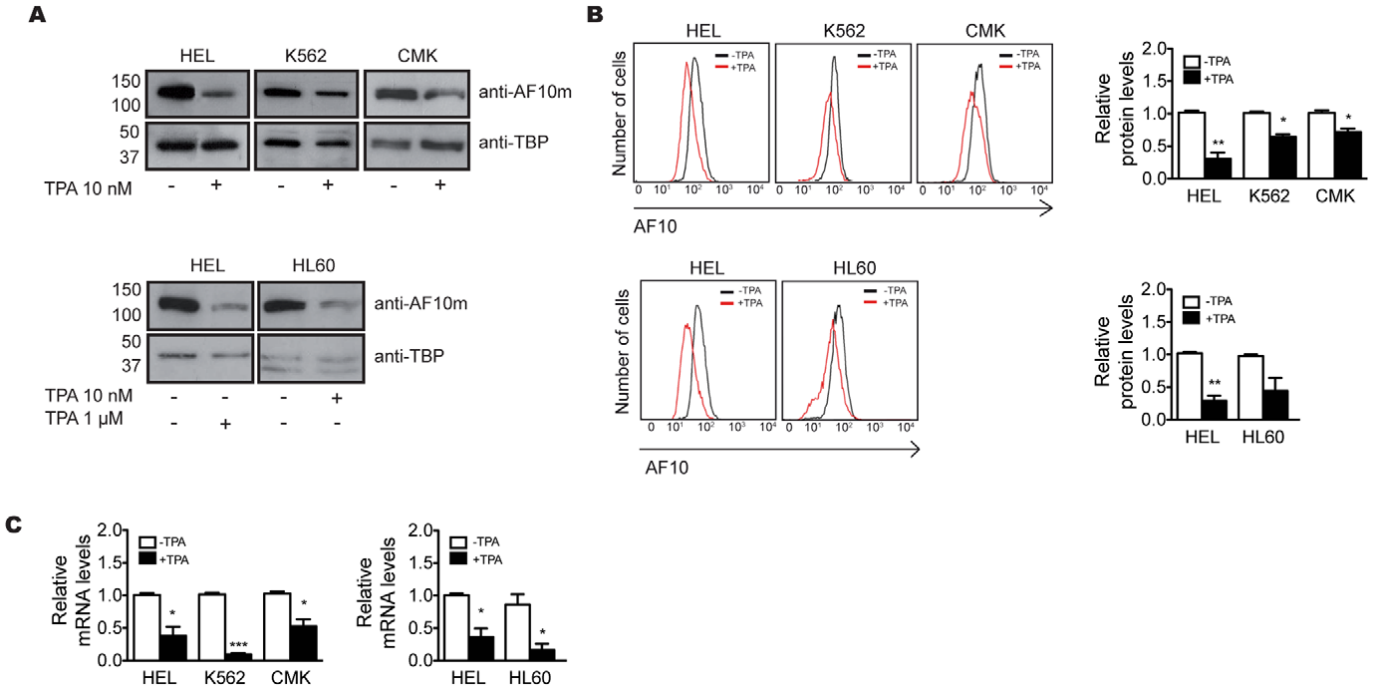
Expression pattern analysis of AF10 protein during hematopoiesis. Detection of AF10 protein by western blot (A) and flow cytometry (B) before (−) and after (+) the treatment with TPA of the cell lines HEL, K562 and CMK, for the megakaryocytic pathway (upper panels), and HEL and HL60, for the monocytic pathway (bottom panels). (C) Relative quantification of the levels of AF10 mRNA by qRT-PCR before (−) and after (+) treatment with TPA. Paired t-test; * P<0.05; ** P<0.01; *** P<0.001 (n≥3).

In order to determine the expression pattern of AF10 during hematopoiesis, these four cell lines were induced to differentiate down the megakaryocytic and/or monocytic pathways by treatment with appropriate concentrations of TPA. At 72 hours post induction, cell cycle and CD41 or F4/80 surface markers levels were analyzed to confirm that the cells were differentiated into megakaryocytes or monocytes, respectively (Figure S2). AF10 protein levels were analyzed by immunofluorescence microscopy and western blot. When compared to the initial levels, both analyses showed a reduction in AF10 protein levels after 72 hours in the presence of TPA irrespective of the cell line or the differentiation pathway (Figure 1A and S3). Intriguingly, HEL, the most undifferentiated of the four cell lines, displayed the highest basal levels of AF10 protein. In order to quantify this decrease, AF10 levels were analyzed by flow cytometry before and after incubation with TPA. As shown in Figure 1B, three of the four cell lines showed a statistically significant (P<0.05) decrease in AF10 protein levels after the induction of differentiation, with the reduction most apparent in the HEL cell line.

We next asked whether this decrease was due to an active mechanism of protein degradation, to the down-regulation of *AF10* expression, or both. The inhibition of the proteasome activity by its specific inhibitor MG123 after hematopoietic differentiation in the HEL cell line led to the maintenance of AF10 levels (Figure S4), indicating that there is an active mechanism of degradation of AF10 upon differentiation. We also analyzed the levels of AF10 mRNA by quantitative real-time RT-PCR (qRT-PCR) before and after the differentiation induction. All four cell lines showed a statistically significant reduction (P<0.05) of mRNA amount after 72 hours of differentiation (Figure 1C). Taken together, these data suggest that AF10 protein levels decrease during hematopoietic differentiation and that this decrease is likely due to the active degradation of the protein through the proteasome and to the diminished AF10 gene expression that results in a reduction in the steady state levels of *AF10* mRNA.

### AF10 overexpression induces cell death in multipotent hematopoietic cells

**Figure 2:**
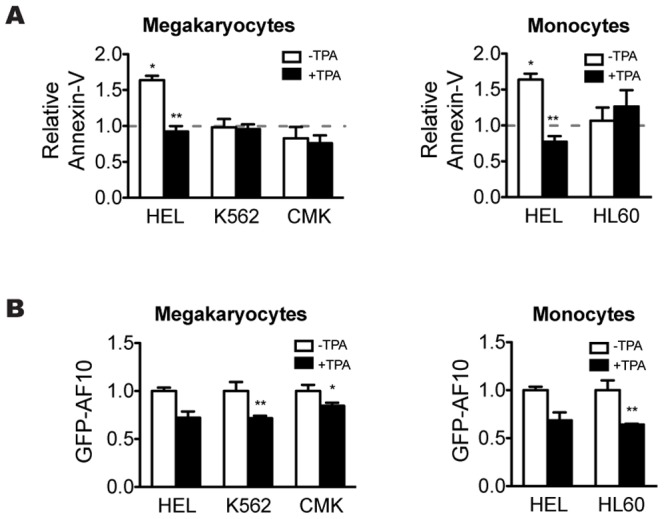
Effect of AF10 over-expression during hematopoiesis. (A) Relative annexin-V in the cell lines transfected with GFP-AF10 plasmid before (−TPA) and after (+TPA) the induction of megakaryocytic differentiation, in cells lines HEL, K562 and CMK, and monocytic differentiation in cell lines HEL and HL60. Dashed line indicates the annexin-V levels of the cells lines transfected with GFP plasmid normalized to 1. (B) Flow cytometry analysis of GFP-AF10 protein levels in all cell lines after the induction of megakaryocytic and monocytic differentiation. Paired t-test; * P<0.05; ** P<0.01 (n≥3).

Since AF10 expression decreased during hematopoietic differentiation, we next asked whether maintenance of proper AF10 mRNA and protein levels was important for this process. First, we tested the effect of AF10 overexpression in stably transfected HEL and K562 cell lines using a tetracycline-regulated promoter to express AF10 or GFP-AF10 fusion proteins (data not shown). The empty vector was used as a negative control. We obtained several stable transfected clones for AF10, GFP-AF10 in the K562 cell line and the empty vector control in both cell lines. However, we were unable to obtain any positive HEL clones expressing AF10 or GFP-AF10 (data not shown). Since positive clones were obtained using the empty vector in the HEL cell line, we reasoned that the lack of HEL clones expressing either transfected AF10 or GFP-AF10 could be due to low levels of AF10 expression due to the known leakiness of the tetracycline inducible promoter. If this were true, then one possible explanation for our inability to identify stable AF10-HEL lines would be that even low levels of AF10 overexpression leads to cell death in the HEL line but not in K562. To test this hypothesis, apoptosis levels were analyzed in all four cell lines transiently transfected with GFP-AF10 or GFP (control). Annexin-V was used as an early apoptotic marker and the GFP signal was used to identify transfected cells. The transfection efficiency varied between 30–75% depending on cell line used (Figure S5). At 18 hours post-transfection, cells were incubated with annexin-V and cell death was quantified by flow cytometry. While no increase in the number of apoptotic cells was observed in K562, HL60 or CMK expressing GFP or GFP-AF10, HEL cells transfected with GFP-AF10 have a statistically significant increase (P<0.01) of the number of annexin-V positive cells as compared to the same cell line transfected with the GFP control (Figure 2A; white bars). This result shows that overexpression of AF10 in HEL cells, but not in any of the other cell lines tested, leads to apoptotic cell death.

Since we found that differentiation leads to reduction of AF10 mRNA and protein levels (Figure 1), we next asked whether this reduction was specific for the endogenous protein. To answer this question, GFP and GFP-AF10 levels were analyzed by flow cytometry in the four cell lines before and after TPA-induced differentiation. As shown in Figure 2B, differentiation induction results in a moderate decrease of relative GFP-AF10 signal in all cell lines irrespective of the differentiation pathway analyzed. GFP-AF10 protein level reduction is less apparent in the HEL cell line. This might be due to the fact that HEL cells display the highest basal AF10 expression levels, hence the decrease of AF10 levels requires longer periods than in the other cell lines. Since transfected GFP-AF10 mRNA levels should not be affected by differentiation induction, the observed decrease in GFP-AF10 protein levels must be mainly due to protein degradation, further supporting the conclusion that induction of differentiation leads to activation of the cellular machinery responsible for the reduction of both AF10 mRNA and protein.

These results led us to predict that induction of differentiation would not only cause a reduction in AF10 protein levels, but would also prevent apoptosis in HEL cells resulting from GFP-AF10 overexpression. To test this hypothesis, we induced the transfected cells to differentiate with TPA down the megakaryocytic and/or monocytic pathway. 18 hours after TPA treatment, apoptosis levels were analyzed by flow cytometry. Induction of differentiation after transfection caused a strong reduction in the number of apoptotic HEL cells irrespective of whether the differentiation pathway followed is megakaryocytic or monocytic (P<0.05) (Figure 2A; black bars). As expected, no changes in annexin-V levels were observed after differentiation induction in any of the other cell lines analyzed. We infer that AF10 levels must be tightly regulated during hematopoietic differentiation.

### AF10 knockdown induces apoptosis in multipotential cells

We next sought to analyze the consequences of reduction in AF10 expression levels, reasoning that if each differentiation stage requires a particular AF10 expression level, it is likely that a strong decrease in AF10 expression might have adverse consequences. Since AF10 expression was shown to be high in the less-committed cells, we hypothesized that a large decrease in AF10 expression would not be tolerated by uncommitted cells. In order to study the effect of knocking down AF10 mRNA, an interference hairpin (siAF10) and the scrambled control siRNA (siControl) were cloned following the GFP cDNA. Since both sequences were expressed in a single mRNA, the GFP signal was used to identify siAF10-expressing cells.

**Figure 3:**
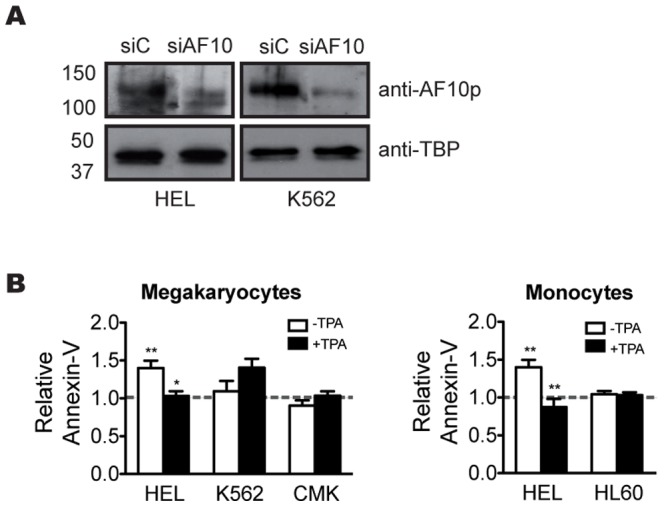
Effect of AF10 loss-of-function during hematopoiesis. (A) Western blot analysis of AF10 expression in HEL and K562 cell lines transfected with scrambled siControl and siAF10. (B) Analysis of annexin-V levels in cell lines transfected with scrambled siControl and siAF10 before (−TPA) and after (+TPA) the induction of megakaryocytic differentiation, in cell lines HEL, K562 and CMK, and monocytic differentiation in cell lines HEL and HL60. Dashed line indicates the annexin-V levels of the cells lines transfected with scrambled siControl plasmid normalized to 1. Paired t-test; * P<0.05; ** P<0.01 (n≥3).

Based in our findings in the overexpression assay, we sought to determine if AF10 knockdown affected cell viability. The four cells lines were transiently transfected with siAF10 and siControl plasmids. 18 hours after transfection, cells were collected and incubated with annexin-V and apoptosis levels measured by flow cytometry. As shown in figure 3A for HEL and K562 cell lines, similar levels of knockdown were achieved in both cell lines. Since cells were transiently transfected and transfection efficiency was estimated between 40 and 80% depending on the cell line (Figure S6), we concluded that the knockdown was effective in the transfected cells. We found that AF10 knockdown in undifferentiated HEL cells led to a significant increase in the number of apoptotic cells (Figure 3B). In contrast, no significant increase was observed in any of the more committed cell lines. This result is in accordance with our hypothesis that AF10 levels must be tightly controlled throughout hematopoietic differentiation in not fully committed cells.

To further confirm our hypothesis, we asked whether the induction of differentiation prevented HEL cells from undergoing apoptosis following AF10 knockdown. After transfection with either siControl or siAF10, megakaryocytic and/or monocytic differentiation was induced in all four cell lines by TPA treatment. Differentiation of HEL cells into megakaryocytes or monocytes resulted in a significant reduction in the number of apoptotic cells as compared with uninduced HEL cells (P<0.05 and <0.01 respectively; Figure 3B). No changes in apoptosis were observed in any of the other cell lines upon differentiation.

### Expression pattern of AF10 in primary cultures from mouse bone marrow

**Figure 4:**
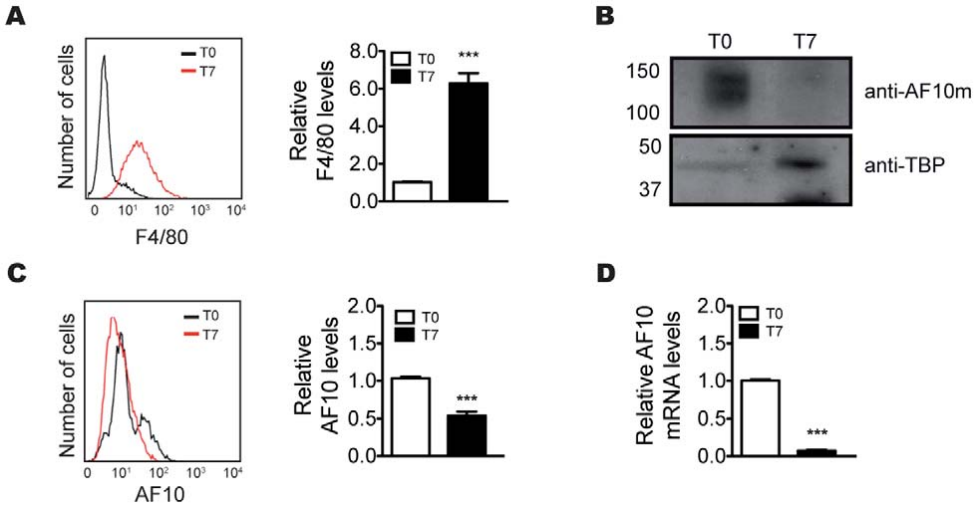
Expression pattern of AF10 in mice bone marrow-derived cells. (A) Quantification of relative F4/80 surface surface expression by flow cytometry before (T0) and after (T7) the cellular differentiation. (B–C) Immunodetection of the protein AF10 by western blot and flow cytometry, respectively, in cells extracted from bone marrow before (T0) and after (T7) the induction of cell differentiation. (D) Quantification by qRT-PCR of the relative levels of AF10 mRNA before (T0) and after (T7) the differentiation of the cells. Paired t-test; *** P<0.001 (n≥3).

The results presented above demonstrating that AF10 levels must be tightly controlled through hematopoietic differentiation led us to hypothesize that AF10 might play an essential role throughout hematopoiesis *in vivo*. To address this question, we performed an *ex vivo* analysis (as a surrogate of *in vivo* experiments) of AF10 expression levels in primary mouse cells during the differentiation process. Hematopoietic cells were isolated from mouse bone marrow and cultured in the presence of M-CSF for 7 days to induce the monocytic pathway. Since F4/80 has been described as a typical monocyte surface marker [39], it was used to monitor the progress of monocytic differentiation induced in the primary cultures. Figure 4A shows that incubation with medium containing M-CSF for 7 days led to a 7-fold increase in F4/80 levels, confirming that monocytic differentiation occurred. To test whether AF10 expression levels were regulated during the monocytic differentiation pathway, AF10 mRNA and protein levels were measured before and after induction of differentiation using western blot and flow cytometric analyses. Primary immature bone marrow cells showed high levels of AF10 protein. In contrast, AF10 protein levels in differentiated monocytes were barely detectable (Figure 4B). A quantitative analysis of AF10 protein expression by flow cytometry revealed a statistically significant reduction (P = 0.001) in its expression in primary bone marrow cells 7 days after induction of differentiation (Figure 4C). We showed above that AF10 mRNA levels are strongly reduced in differentiated cells irrespective of the differentiation pathway studied (Figure 1D). To further investigate the relevance of these data, we measured AF10 mRNA levels in primary bone marrow cells and differentiated monocytes. AF10 expression was reduced more than 90% after 7 days of differentiation, confirming that AF10 expression is strongly regulated in primary cultures of hematopoietic cells during the hematopoietic differentiation (Figure 4D). In summary, our results demonstrate that AF10 expression is tightly regulated through hematopoietic differentiation, suggesting that it plays an important role in the process.

## Discussion

In this study, we examined the expression pattern of transcription factor AF10 during hematopoietic differentiation through the megakaryocytic and monocytic pathways. The *in vitro* assays developed in the human hematopoietic cell lines HEL, K562, CMK and HL60 showed a reduction of both AF10 protein and mRNA levels during the progression of cell differentiation (Figure 1-S3), suggesting an *in vivo* down regulation of *AF10* in the later stages of hematopoiesis. A detailed analysis of these results revealed that the four cell lines used in this work (all of which are at different stages of hematopoietic differentiation), expressed different basal AF10 levels before differentiation. HEL, the most “uncommitted” cell line in our panel, expressed the most AF10 before differentiation (Figure S3). The AF10 abundance in the other three undifferentiated cell lines correlates with their respective commitment in the hematopoietic lineage (Figure S3). Accordingly, the analysis of differentiated cells overexpressing AF10 shows a distinct reduction in protein levels (Figure 2A). As AF10 overexpression is driven by a viral promoter, and therefore is not developmentally regulated, this result, together with maintenance of AF10 protein levels in differentiated cells after proteasome inhibition (Figure S4), shows that the decrease in protein levels is likely due to active protein degradation, and not just transcriptional down-regulation. This indicates that both *AF10* gene silencing and active protein degradation mechanisms contribute to its regulation. Taken together, these results suggest that AF10 levels must be tightly regulated throughout hematopoietic differentiation. To determine whether the results obtained in cell lines reflect the *in vivo* role of AF10, we analyzed AF10 in primary cells. Monocytic differentiation was induced in immature hematopoietic cells from mouse bone marrow and the levels of both AF10 protein and mRNA were measured before and after differentiation. We observed a strong reduction of AF10 protein and mRNA (Figure 4), supporting the premise that AF10 plays role during hematopoiesis *in vivo*.

Recent reports by *Mahmoudi et al.* and *Mohan et al*. showed that in mice, zebrafish and Drosophila, Dot1L, Tcf4/β-catenin and AF10 form a stable complex along with several other proteins [31], [32]. Dot1L is a well-characterized histone methyltransferase whose interaction with AF10 has long been established [20]. Although there are no *in vivo* data available on the role of AF10 during hematopoiesis, it has been recently shown that *Dot1L* ^−/−^ mice and embryonic stem cells have severe defects in hematopoietic differentiation due to the block of cell-cycle progression which promotes apoptosis of hematopoietic progenitor cells [40], [41]. β-catenin is key in the Wnt pathway, which serves a critical role in tissue development, progenitor cell proliferation, and many human cancers [42]. Canonical Wnt signaling is considered to be an important pathway for maintaining or inducing an undifferentiated phenotype in HSCs [43], [44]. Therefore, over-expression of Wnt signaling components can potentially enhance HSC self-renewal. In contrast, loss of canonical Wnt signaling may lead to impaired self-renewal, showing that maintenance of Wnt signaling at the exact dose is critical for HSC function [42]. This pathway has also been shown to regulate apoptosis in many tissues, and activation of canonical Wnt signaling has been associated with either a decrease or increase in apoptosis [45], [46]. Considering this information, the presence of Tcf4/β-catenin in the AF10-Dot1L complex also supports an *in vivo* role of AF10 during hematopoietic differentiation.

In agreement with these observations, the data obtained from the AF10 gain and loss of function experiments show that these alterations induce cell death by apoptosis in the more uncommitted cell line HEL, but not in any of the other cell lines analyzed (Figures 2–3). Moreover, induction of differentiation in HEL cells rescues cell death induced by both AF10 over-expression or knockdown (Figures 2B–3B). Considering that HEL cells are the only cells used in these experiments that maintain multipotential characteristics, these results indicate that the alteration of AF10 levels compromises the viability of uncommitted cells. Accordingly, the loss of uncommitted cell properties due to the differentiation process prevents cell death induction by changes in AF10 levels (Figures 3A–4A). Since the catalytic properties of Dot1L are regulated *in vivo* by AF10 [32], it is key that *Dot1L* ^−/−^ embryonic stem cells present elevated apoptosis levels and G2 cell cycle arrest in hematopoietic progenitor cells [41], [47]. These data agree with gene expression studies showing that in *Dot1L^−/−^* cells, gene ontology groups related to cell proliferation, cell cycle progression, and apoptosis are statistically overrepresented in a list of differentially expressed RNAs [48]. Taken together, these results support our hypothesis that precise control of AF10 levels is crucial in early stages of hematopoiesis.

In conclusion, we have demonstrated the significance of AF10 during the uncommitted stages of hematopoiesis, as well as the importance of AF10 maintenance at these stages. Based on these findings, we infer that AF10 could be directly or indirectly implicated in cell cycle regulation. Changes in expression levels leads to apoptosis only in the multipotential cells, which retain proliferative capacity. AF10's interaction with Ikaros, a protein involved in lymphocyte cell cycle control [26], [29], as well as with Dot1L and β-catenin, both of which are involved in the regulation of apoptosis and hematopoiesis [32], supports our findings. Although further investigation is needed to reveal the molecular mechanisms of AF10 action during hematopoiesis, our data demonstrate that AF10 participates in the early stages of hematopoiesis and contribute to a better understanding of the link between the translocation of *AF10* and the development of lymphoid and myeloid proliferative diseases.

## Supporting Information

Figure S1 **Schematic representation of protein structures and model system. Schematic representation of AF10, MLL-AF10 and CALM-AF10 structure.** Arrows show the break points in AF10 when the chromosomal rearrangements occur. OM: octapeptide motif; LZ: leucine zipper.
(TIF)

Figure S2 **Cell line differentiation.** (**A**) **DNA content in HEL, K562, CMK and HL60 cell lines before and after TPA treatment for the induction of megakaryocytic (upper panels) or monocytic (bottom panels) differentiation.** (B) Levels of specific markers of megakaryocytic (CD41) and monocytic (F4/80) differentiation before and after the exposure to TPA. Paired t-test; * P<0.05; ** P<0.01 (n≥3).
(TIF)

Figure S3 **Immunohistochemistry.** AF10 immunodetection (green) in the cell lines HEL, K562, CMK and HL60 before (−TPA) and after (+TPA) megakaryocytic (A) or monocytic (B) differentiation. DNA was stained with DAPI (blue).
(TIF)

Figure S4 **Inhibition of the proteasome activity during differentiation.** Western blot analyses of AF10 levels in the cell line HEL. Cells were incubated with TPA for 24 h followed by 24 h of treatment with the specific proteasome inhibitor MG132 or DMSO (vehicle). AF10 analyses were performed in cells exposed to DMSO (1^st^ lane), to TPA during 24 h or 48 with TPA (2^nd^ and 3^rd^ lanes respectively) and 48 h with TPA and the last 24 h also with MG123 (4^th^ lane). AF10 was detected with antibody anti-AF10m and TBP was used as loading control.
(TIF)

Figure S5 **Overexpression efficiency determination by flow cytometry.** Untransfected cells (left panels) were assayed to determine the threshold for GFP detection. Cells transfected with GFP and GFP-AF10 plasmids were analyzed to determine the transfection efficiency. Percentages indicate the percentage of GFP positive cells.
(TIF)

Figure S6 **Determination of knockdown efficiency by flow cytometry.** Untransfected cells (left panels) were assayed to determine the threshold for GFP detection. GFP signal after transfection with siControl or siAF10 plasmids represents the transfected population. Percentages indicate the percentage of GFP positive cells.
(TIF)

Table S1 **Oligonucleotides sequences used for AF10 knock-down.** In red, complementary regions that will generate the dsRNA. The underlined sequence indicates the sequence that will recognize AF10 mRNA region that will activate the dsRNA degradation machinery. siAF10, interference plasmid; siControl, control plasmid with no homology to any known vertebrate DNA sequence.
(XLSX)
