# Supplementary figures and images for: AF10 Plays a Key Role in the Survival of Uncommitted Hematopoietic Cells
Source: PLoS One. 2012 Dec 19;7(12):e51626. doi: 10.1371/journal.pone.0051626 (PMC3526614; doi:10.1371/journal.pone.0051626)

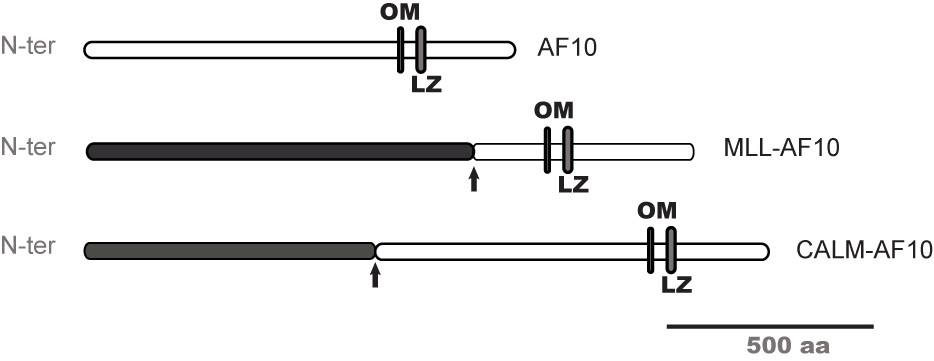

Supplement: Figure S1 — Schematic representation of protein structures and model system. Schematic representation of AF10, MLL-AF10 and CALM-AF10 structure. Arrows show the break points in AF10 when the chromosomal rearrangements occur. OM: octapeptide motif; LZ: leucine zipper. (TIF) [file pone.0051626.s001.tif]

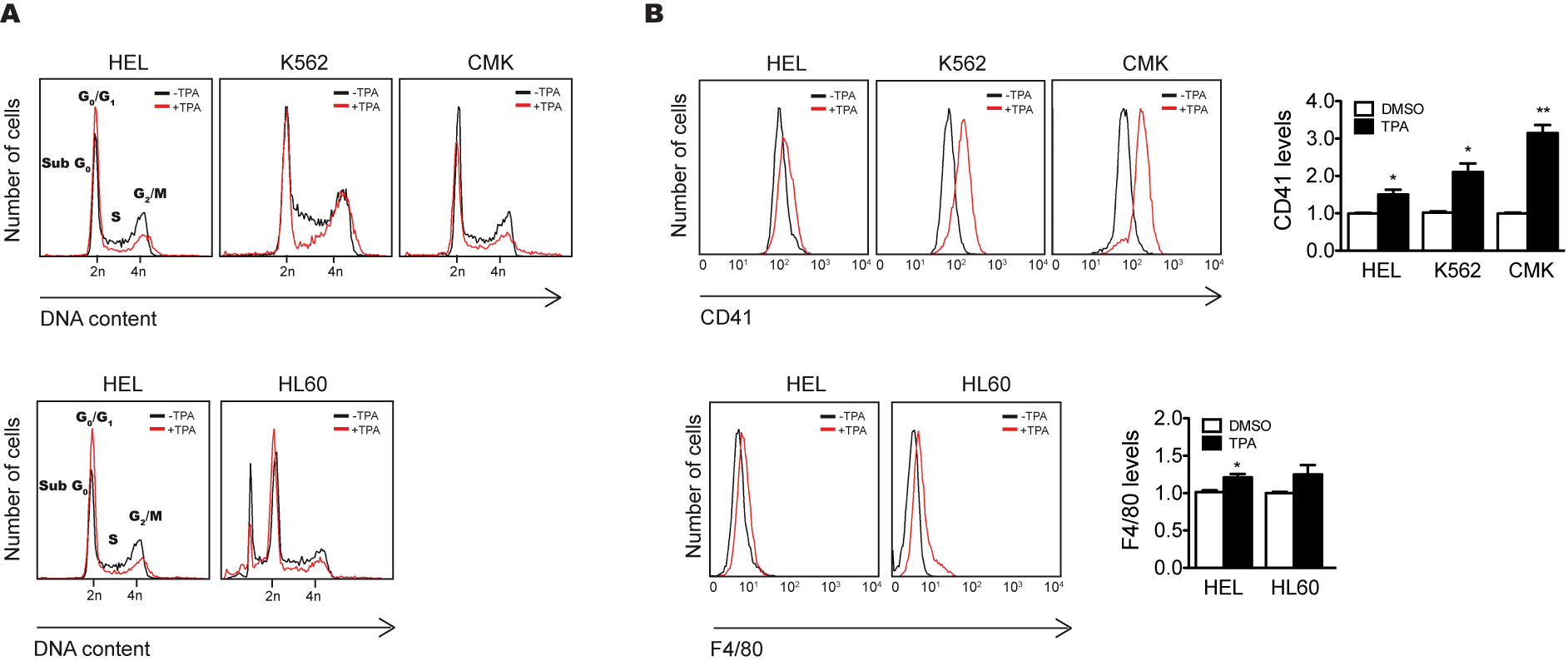

Supplement: Figure S2 — Cell line differentiation. (A) DNA content in HEL, K562, CMK and HL60 cell lines before and after TPA treatment for the induction of megakaryocytic (upper panels) or monocytic (bottom panels) differentiation. (B) Levels of specific markers of megakaryocytic (CD41) and monocytic (F4/80) differentiation before and after the exposure to TPA. Paired t-test; * P<0.05; ** P<0.01 (n≥3). (TIF) [file pone.0051626.s002.tif]

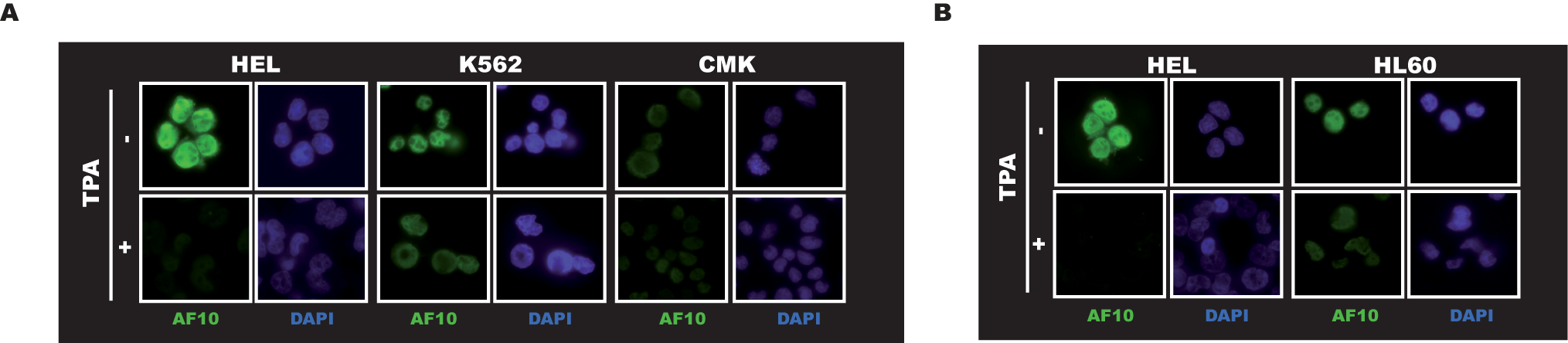

Supplement: Figure S3 — Immunohistochemistry. AF10 immunodetection (green) in the cell lines HEL, K562, CMK and HL60 before (−TPA) and after (+TPA) megakaryocytic (A) or monocytic (B) differentiation. DNA was stained with DAPI (blue). (TIF) [file pone.0051626.s003.tif]

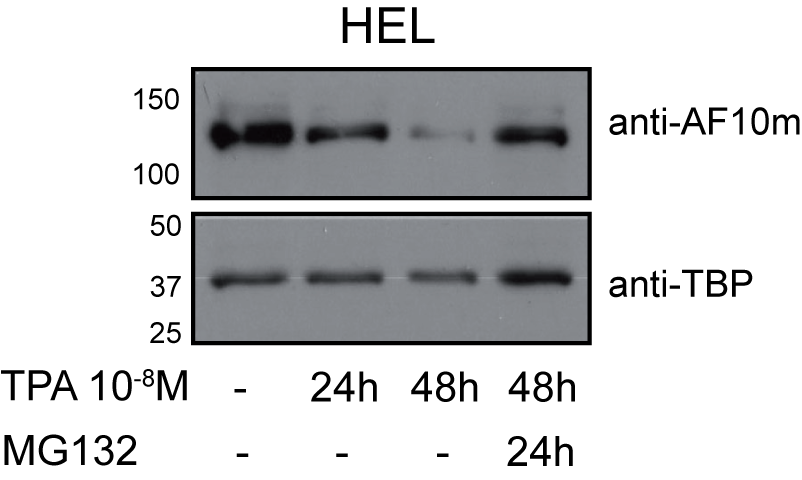

Supplement: Figure S4 — Inhibition of the proteasome activity during differentiation. Western blot analyses of AF10 levels in the cell line HEL. Cells were incubated with TPA for 24 h followed by 24 h of treatment with the specific proteasome inhibitor MG132 or DMSO (vehicle). AF10 analyses were performed in cells exposed to DMSO (1st lane), to TPA during 24 h or 48 with TPA (2nd and 3rd lanes respectively) and 48 h with TPA and the last 24 h also with MG123 (4th lane). AF10 was detected with antibody anti-AF10m and TBP was used as loading control. (TIF) [file pone.0051626.s004.tif]

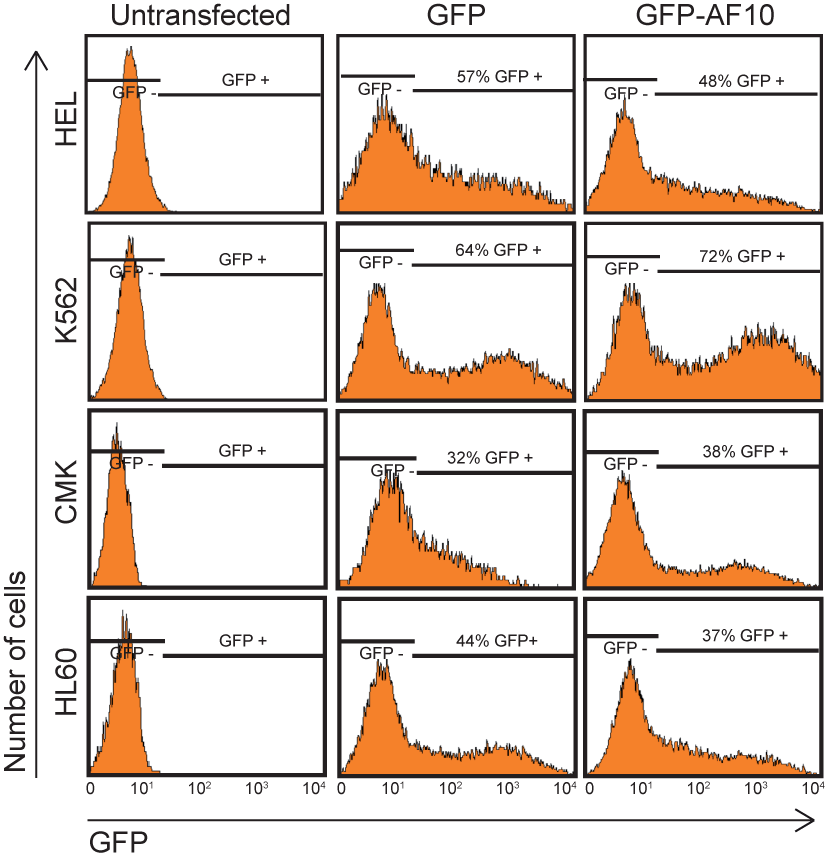

Supplement: Figure S5 — Overexpression efficiency determination by flow cytometry. Untransfected cells (left panels) were assayed to determine the threshold for GFP detection. Cells transfected with GFP and GFP-AF10 plasmids were analyzed to determine the transfection efficiency. Percentages indicate the percentage of GFP positive cells. (TIF) [file pone.0051626.s005.tif]

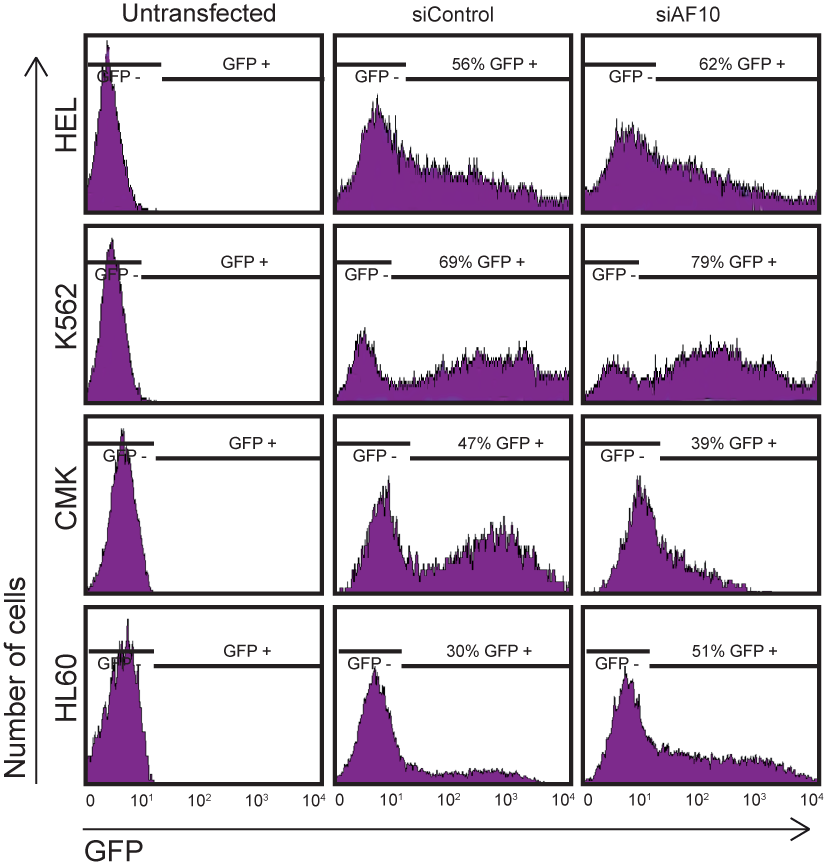

Supplement: Figure S6 — Determination of knockdown efficiency by flow cytometry. Untransfected cells (left panels) were assayed to determine the threshold for GFP detection. GFP signal after transfection with siControl or siAF10 plasmids represents the transfected population. Percentages indicate the percentage of GFP positive cells. (TIF) [file pone.0051626.s006.tif]
